# The Potential Utility of Curcumin in the Treatment of HER-2-Overexpressed Breast Cancer: An *In Vitro* and *In Vivo* Comparison Study with Herceptin

**DOI:** 10.1155/2012/486568

**Published:** 2011-06-06

**Authors:** Hung-Wen Lai, Su-Yu Chien, Shou-Jen Kuo, Ling-Ming Tseng, Hui-Yi Lin, Chin-Wen Chi, Dar-Ren Chen

**Affiliations:** ^1^Comprehensive Breast Cancer Center, Changhua Christian Hospital, Changhua 50006, Taiwan; ^2^Department and Institute of Pharmacology, School of Medicine, National Yang-Ming University, Taipei 11221, Taiwan; ^3^Department of Pharmacology, Changhua Christian Hospital, Changhua 50006, Taiwan; ^4^School of Medicine, College of Health Care and Management, Chung Shan Medical University, Taichung 40201, Taiwan; ^5^School of Nutrition, College of Health Care and Management, Chung Shan Medical University, Taichung 40201, Taiwan; ^6^Division of General Surgery, Department of Surgery, Taipei Veterans General Hospital, Taipei 11217, Taiwan; ^7^School of Medicine, National Yang-Ming University, Taipei 11221, Taiwan; ^8^Department of Pharmacology, School of Pharmacology, China Medical University, Taichung 40402, Taiwan

## Abstract

HER-2 is an important oncoprotein overexpressed in about 15–25% of breast cancers. We hypothesized that the ability of curcumin to downregulate HER-2 oncoprotein and inhibit the signal transduction pathway of PI3K/Akt, MAPK, and NF-*κ*B activation may be important in the treatment of HER-2-overexpressed breast cancer. To examine the effect of curcumin on breast cancer cells, MCF-7, MDA-MB-231, MCF-10A, BT-474, and SK-BR-3-hr (a herceptin resistant strain from SK-BR-3) cells were used for *in vitro* analysis. The *in vivo* effect of curcumin on HER-2-overexpressed breast cancer was investigated with the HER-2-overexpressed BT-474 xenograft model. Cell growth, cell cycle change, the antimobility effect, signal transduction, and xenograft volume analysis between groups treated with herceptin and/or curcumin were tested. Curcumin decreased the cell growth of various breast cancer cell lines (MCF-7, MDA-MB-231, MCF-10A, BT-474, and SK-BR-3-hr). In Western blot analysis, the phosphorylation of Akt, MAPK, and expression of NF-*κ*B were reduced in BT-474 cells, but not in SK-BR-3-hr cells, after treatment with herceptin. When treated with curcumin, the HER-2 oncoprotein, phosphorylation of Akt, MAPK and expression of NF-*κ*B were decreased in both BT-474 and SK-BR-3-hr cells. In the BT-474 xenograft model, though not as much as herceptin, curcumin did effectively decrease the tumor size. The combination of curcumin with herceptin was not better than herceptin alone; however, the combination of taxol and curcumin had an antitumor effect comparable with taxol and herceptin. The results suggested that curcumin has potential as a treatment for HER-2-overexpressed breast cancer.

## 1. Introduction

Around 15–25% of breast cancers are noted to overexpress the human epithelial growth factor receptor 2 (HER-2) [1–3], patients with HER-2 overexpression were associated with a poor prognosis, more disease relapse, and distant metastasis [3–6]. Herceptin (generic name: Trastuzumab), which effectively inhibits the HER-2-related PI3k/Akt and MAPK pathways, is the first targeted therapeutic agent developed and approved for the treatment of patients with HER-2-overexpressed breast cancer [7–12]. Despite the success of herceptin, a significant proportion of HER-2-positive breast cancer patients responded poorly to the treatment [13, 14]. In addition, some patients that initially responded to the therapy became resistant within one year [14, 15]. The refractory state of these HER-2-positive breast carcinomas illustrates the need to examine the mechanisms underlying tumor resistance and the necessity to seek novel treatment strategies.

Curcumin (diferuloylmethane) is a yellow pigment derived from the rhizome of the plant *Curcuma longa L*. The powdered rhizome of this plant, called turmeric, is commonly used in the preparation of curries. Curcumin, as a polyphenol with a diarylheptanoid structure containing two *α*, *β*-unsaturated ketones, is considered to be the major active constituent of turmeric [16, 17]. In addition to its wide range of pharmacological activities, the anticancer properties of curcumin have attracted great interest [18–20]. The ability of curcumin to downregulate EGFR [19, 21, 22] and HER-2 [23] oncoproteins and affect the PI3K/Akt [24] and MAPK [25] pathways, with which herceptin interfered, raised interest in the potential utility of curcumin in the treatment of HER-2-positive breast cancer.

Taxol (generic name: Paclitaxel) combined with herceptin is one of the current preferred regimens for the treatment of HER-2-overexpressed breast cancer [11, 26]. NF-*κ*B is an important factor related to proliferation and antiapoptosis [27, 28], and the activation of NF-*κ*B plays an important role in the chemoresistance of taxol [29, 30]. Curcumin has the well-known ability to downregulate NF-*κ*B [31, 32]. The combination of curcumin with taxol could suppress taxol-related NF-*κ*B activation and enhance the antitumor effect of taxol [31, 33]. The evolving concept of combining a monoclonal antibody (herceptin or pertuzumab) for the extracellular domain of HER-2 and small molecule tyrosine kinase inhibitors (TKI) for EGFR (gefitinib, erlotinib) [34–36] or EGFR/HER-2 (lapatinib) [37] has shown benefit in some preclinical studies and patient trials. The multifunctions of curcumin in downregulating EGFR and HER-2 oncoproteins, reducing the phosphorylation of Akt and MAPK and suppressing NF-*κ*B activation, led to interest in using curcumin in the treatment of HER-2-overexpressed breast cancer, along with herceptin and/or taxol.

The aim of this preclinical study was to explore the potential application of curcumin in the treatment of HER-2-overexpressed breast cancer and examine the interaction of curcumin and herceptin, which has rarely been reported.

## 2. Materials and Methods

### 2.1. Cell Culture and Reagents

**Figure 1:**
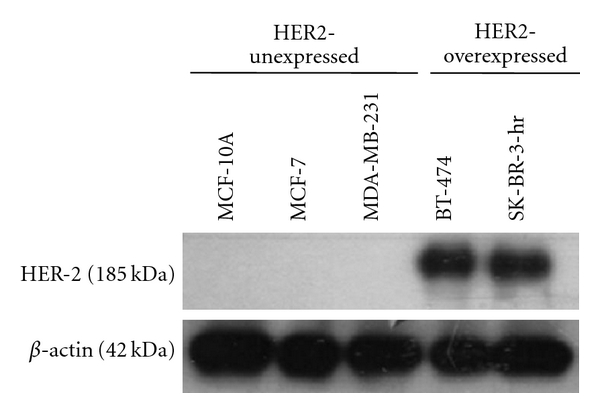
The expression of HER-2 oncoprotein in various human breast cancer cell lines. Cells were lysed and analyzed by western blot as described in methods. As revealed in figure, BT-474 and SKBr-3-hr were HER-2-overexpressed breast cancer cell lines. Whiles MCF-7, MCF-10A, and MDA-MB-231 cells were HER-2-unexpressed breast cancer cells.

To examine the effect of herceptin and curcumin on breast cancer cell lines with various estrogen receptors (ER) and HER-2 receptors, MCF-7 [ER(+), HER-2(−)], SK-BR-3-hr (a herceptin-resistant strain from SK-BR-3 breast cancer cells) [ER(−), HER-2(+)], BT-474 [ER(+), HER-2(+)], MDA-MB-231 [ER(−), HER-2(−)], and normal breast epithelial cells, MCF-10A [ER(−), HER-2(−)], were chosen. The expression of HER-2 oncoprotein in MCF-7, MCF-10A, MDA-MB-231, BT-474, and SK-BR-3-hr cells was verified by western blotting (Figure 1).

The cell lines (MCF-7, BT-474, MDA-MB-231, and normal breast MCF-10A) used in this study were purchased from American Type Culture Collection (ATCC). SK-BR-3-hr, a herceptin-resistant strain, was a kind gift from Dr. L.-M. Tseng. The resistance of herceptin was induced by repeated culture of herceptin-treated SK-BR-3 cells, which were herceptin-sensitive and purchased from ATCC. The MCF-7 cell line was maintained in RPMI medium with 10% fetal bovine serum, 50 unit/mL penicillin, and 50 unit/mL streptomycin. The MDA-MB-231 cells were cultured in L15 medium containing 10% fetal bovine serum, 50 unit/mL penicillin, and 50 unit/mL streptomycin. BT-474 cells were maintained in DMEM/F12 medium with 10% fetal bovine serum, 50 unit/mL penicillin, and 50 unit/mL streptomycin. SK-BR-3-hr cells were maintained in DMEM/F12 medium with 10% fetal bovine serum, 50 unit/mL penicillin, and 50 unit/mL streptomycin. MCF-10A was maintained in MEBM medium with 1% penicillin-streptomycin, 50 *μ*g/mL hydrocortisone, 1 *μ*g/mL EGF, 500 *μ*g/mL insulin, and 10 *μ*g/mL cholera toxin. Cells were maintained at 37°C in a humidified atmosphere in the presence of 5% CO_2_.

### 2.2. Compounds

Curcumin (Sigma-Aldrich, Inc., St. Louis, Mo, USA) was dissolved in DMSO at 50 *μ*g/mL as a stock solution. Herceptin was purchased from Roche and dissolved in PBS at 50 *μ*g/mL as stock. Taxol was dissolved in 49.7% ethanol at 6 mg/mL as stock.

### 2.3. Growth and Cell Proliferation Analysis

Cell proliferation was measured using sulforhodamine B (SRB) colorimetric analysis, which is used for cell density determination, based on the measurement of cellular protein content [38]. The method described here has been optimized for the toxicity screening of compounds to adherent cells in a 96-well format. After an incubation period, cell monolayers were fixed with 10% (wt/vol) trichloroacetic acid and stained with SRB for 30 min after which the excess dye was removed by washing repeatedly with 1% (vol/vol) acetic acid. The protein-bound dye was dissolved in 10 mM Tris base solution for OD determination at 510 nm using a microplate reader. The results were linear over a 20-fold range of cell numbers.

The trypan blue exclusion test was also used to confirm the cell proliferation result in herceptin- and curcumin-treated BT-474 and SK-BR-3-hr cells. Cells were stained with 4% trypan blue (Sigma), and viable cells counted with a hemocytometer under a light microscope.

### 2.4. Determination of Combinatorial Effects

The ability of herceptin and curcumin to act in a synergistic, additive, or antagonistic matter with regard to growth inhibition was determined by a combination index (CI) as proposed by Chou and Talalay [39, 40]. The Calcusyn software (Biosoft, Great Shelford, Cambridge, UK) was used to determine the CI for each concentration of drug mixture used. A CI value <1 represented a case where synergism of herceptin and curcumin was present. CI values of 1 and >1 represented additive and antagonistic effects, respectively.

### 2.5. Cell Cycle Analysis

Breast cancer cells were plated at a density of 5 × 10^5^/dish in 60 × 15-mm culture dishes and allowed to adhere overnight. Cells were then incubated with media containing herceptin and/or curcumin at different concentrations as indicated. Following 72 hours (h) of incubation, the cells were washed with PBS, trypsinized, and collected by centrifugation at 1,500 rpm for 10 minutes. After centrifugation, the supernatant was removed, and the cell pellets were fixed with 75% alcohol at −20°C. After 1 h of incubation, the cell pellets were collected by centrifugation at 1,500 rpm for 10 minutes. The pellets were incubated with propidium iodide (PI) solution (10 *μ*g/mL) for 30 minutes. The cell cycle phase was determined by Cytomics FC500 flow cytometry (Beckman Coulter).

### 2.6. Mobility Test

Cell migration is necessary in many physiological processes, such as wound healing, and is a characteristic of cancer cell metastasis [41]. The inhibitory effect of herceptin and/or curcumin on different breast cancer cell lines was tested by wound healing assay. Upon inflicting a scratch wound, these cells were treated with various concentrations of curcumin and/or herceptin for 4 h and returned to standard media in an attempt to minimize any cytotoxic effects that could potentially confound our observations. Following 20 h of further incubation, the areas of the wounds were measured using Image J software (http://rsb.info.nih.gov/ij/).

### 2.7. Western Blot Analysis

Breast cancer cells were allowed to incubate with curcumin and/or herceptin at various concentrations and time points as indicated and harvested after treatment. Whole cell lysate was prepared by resuspending the cells in RIPA buffer supplemented with protease inhibitors cocktail (PIERCE) and incubating the cells on ice for 30 minutes. Cell lysates were centrifuged at 13,000 x g for 10 minutes and the supernatant collected. The protein concentration was measured using the Bradford assay (Bio-Rad Laboratories, Hercules, Calif, USA).

An aliquot of protein lysate (10 *μ*g) from each sample was mixed with 2X Laemmli sample buffer (Bio-Rad, Hercules, Calif, USA), and the protein lysate was separated in 10% SDS-polyacrylamide gels for 1 h. After transferring the sample to a nitrocellulose membrane, the membrane was blocked with 5% milk in 1X TBST buffer (10 mM Tris, 150 mM NaCl, 0.5% Tween-20, pH 7.4) for 1 h at room temperature and immunoblotted using the following antibodies: HER-2, phosphorylated Akt (p-Akt), total Akt, phosphorylated MAPK (p-MAPK), total MAPK, and NF-*κ*B (Cell Signaling Technology). The proteins were probed with anti-HER-2 (Cell Signaling Technology), anti-p-Akt, anti-total Akt, anti-p-ERK1/2, anti-ERK 1/2, anti-NF-*κ*B, and anti-*β*-actin (Sigma) at 4°C overnight, followed by incubation with horseradish peroxidase-conjugated secondary antibodies (Sigma). Protein visualization was performed using the enhanced chemiluminescence kit (PIERCE) according to the manufacturer protocol. Equal loading of total protein was normalized with the *β*-actin signal.

### 2.8. In Vivo Model: Xenograft of HER-2-Overexpressed Breast Cancer in Nude Mice

HER-2-overexpressed BT-474 cells (1 × 10^7^ cells per mice) were injected into 4- to 6-week-old female, athymic nude mice subcutaneously (s.c.) in the right flank region to form xenografts. Prior to tumor cell inoculation, all mice were primed with 17*β*-estradiol pellets introduced subcutaneously in a biodegradable carrier binder 7 days before inoculation of the tumor (1.7 mg of estradiol per pellet, Innovative Research of America, Inc.) to promote tumor growth. Then, 1 × 10^7^ BT-474 cells, suspended in (200 *μ*L) growth-factor-reduced matrigel (BD Bioscience, Bedford, MA), were injected s.c. into the right flank region. A period of 14 to 21 days elapsed to allow formation of tumor nodules. Tumor nodules were monitored twice weekly by a single observer using serial micrometer (mm) measurements, with tumor volume calculated as the product of length × width^2^/2. Six animals were randomly assigned to each treatment group. Statistical tests were performed to assure uniformity in starting volumes between treatment and control groups at the beginning of each experiment.

Treatment groups included the control, herceptin alone, curcumin alone, and the combination of herceptin and curcumin. To evaluate the effect of curcumin and/or herceptin with chemotherapeutic agents, taxol alone, taxol and herceptin, taxol and curcumin, and taxol + herceptin + curcumin regimens were tested. Treatment with different protocols was initiated 21–28 days postxenograft inoculation status, at which time the xenograft volume was measured at around 50–100 mm^3^. Differences in xenograft volume on the 28th posttreatment day between groups were analyzed by single-factor analysis of variance (ANOVA). The treatment protocol is summarized.

- Control group: sterile 0.1% DMSO intra-peritoneally (i.p.) injection once per week for 4 consecutive weeks.
- Herceptin-only group: loading dose of 4 mg/kg herceptin in sterile PBS, administered by i.p. injection, then a dose of 2 mg/kg maintained at once per week, for 4 consecutive weeks.
- Curcumin only: curcumin dissolved in 0.1% DMSO injected i.p. at a dose of 45 mg/kg twice per week for 4 consecutive weeks.
- Combined curcumin and herceptin: curcumin dissolved in 0.1% DMSO injected i.p. at a dose of 45 mg/kg twice per week, combined with a loading dose of 4 mg/kg herceptin in sterile PBS, administered by i.p. injection, then a dose of herceptin 2 mg/kg maintained at once per week, for 4 consecutive weeks.
- Taxol-alone group: taxol 10 mg/kg i.p. once per week for 4 consecutive weeks.
- Taxol + herceptin: taxol 10 mg/kg i.p. once per week, combined with a loading dose of 4 mg/kg herceptin in sterile PBS, administered by i.p. injection, then a dose of herceptin 2 mg/kg maintained at once per week, for 4 consecutive weeks.
- Taxol and curcumin: taxol 10 mg/kg i.p. once per week, curcumin dissolved in DMSO injected i.p. at a dose of 45 mg/kg twice per week for 4 consecutive weeks.
- Taxol, curcumin, and herceptin: taxol 10 mg/kg i.p. once per week, curcumin dissolved in 0.1% DMSO injected i.p. at a dose of 45 mg/kg twice per week, combined with a loading dose of 4 mg/kg herceptin in sterile PBS, administered by i.p. injection, then a dose of herceptin maintained at 2 mg/kg once per week, for 4 consecutive weeks.

After completing 4 weeks of treatment, the mice were sacrificed before recording body weight and tumor volume. The tumor was harvested and processed for various analytical purposes. The animal use protocol were reviewed and approved by the Institutional Animal Care and Use Committee of Changhua Christian Hospital, Changhua, Taiwan.

### 2.9. Statistical Analysis

Analyses were performed using the Statistical Analysis System (SAS 9.1). Data are presented as mean ± standard deviation, except where indicated. Comparisons between groups were analyzed using the chi-square test, Student's two-tailed *t*-test, or one-way ANOVA with Bonferroni's correction, as appropriate. A value of *P* < 0.05 is considered statistically significant.

## 3. Results

### 3.1. Effects of Herceptin or Curcumin on Cell Proliferation

**Figure 2:**
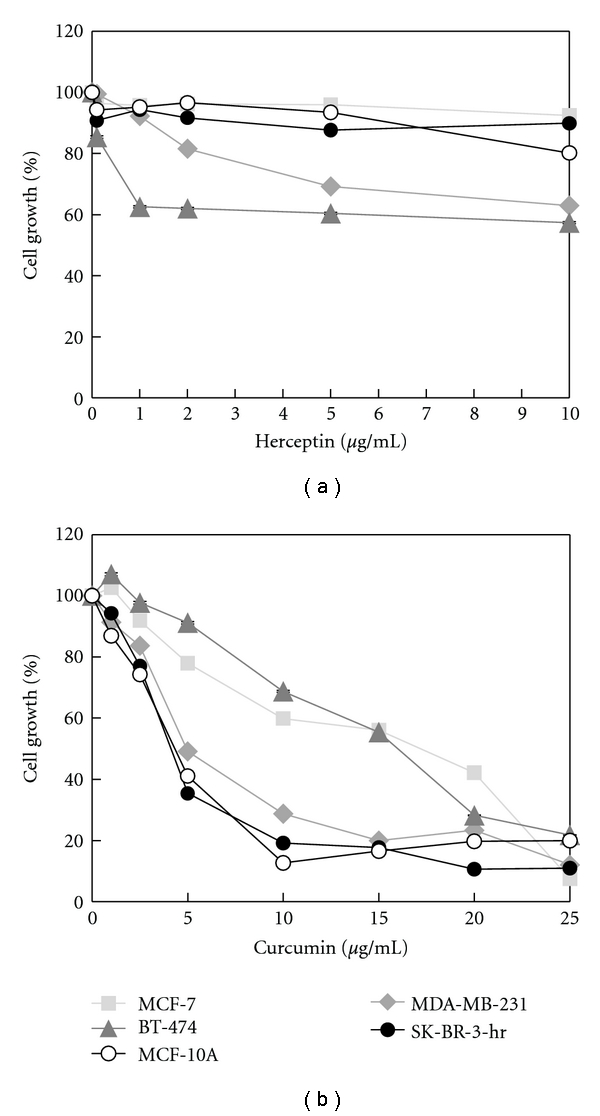
The effect of herceptin and curcumin on growth of different breast cancer cell lines. Cells were incubated with different levels of herceptin (a) or curcumin (b) for 72 h, respectively, and cell growth was measured using SRB assay.

To examine the biological effect of herceptin and curcumin, breast cancer cell lines were treated with different concentrations of herceptin (0.1–10 *μ*g/mL) or curcumin (1–25 *μ*g/mL) for 72 h. Cell proliferation change was assayed with an SRB assay. As shown in Figure 2(a), in HER-2-overexpressed BT-474 breast cancer cells, cell proliferation was inhibited by herceptin in a dose-dependent manner. Cell proliferation decreased to 60% after treatment with 1 *μ*g/mL of herceptin and reached a plateau when with a concentration >1 *μ*g/mL. In SK-BR-3-hr breast cancer cells, an HER-2-overexpressed and herceptin-resistant breast cancer cell line, cell growth was not inhibited by herceptin, even at a 10 *μ*g/mL high concentration. The growth of MCF-7 and MCF-10A, which were non-HER-2-overexpressed cells, was not affected by herceptin treatment. The growth of MDA-MB-231 cells decreased to 64% of the control after 10 *μ*g/mL of herceptin treatment (Figure 2(a)).

In examining the effect of curcumin treatment on these cell lines, we found that the cell proliferations of these five cell lines (MCF-7, BT-474, SK-BR-3-hr, MCF-10A, and MDA-MB-231) were all decreased after treatment with curcumin, with different sensitivities (Figure 2(b)). The SK-BR-3-hr, MCF-10A, and MDA-MB-231 cells were more sensitive to curcumin than BT-474 and MCF-7 cells. After treatment with 10 *μ*g/mL of curcumin, the cell proliferation of HER-2-overexpressed BT-474 cells and herceptin-resistant SK-BR-3-hr cells decreased to 65% and 20%, respectively.

### 3.2. Combination Treatment of Cells with Herceptin and Curcumin

**Figure 3:**
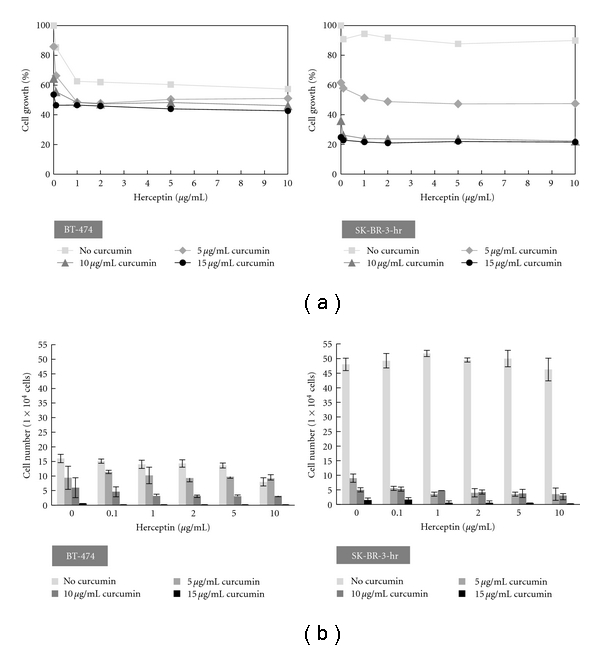
Cell growth of HER-2-overexpressed breast cancer cells with combined treatment of herceptin and curcumin. (a) The combined effects of herceptin and curcumin on the growth of BT-474 and SK-BR-3-hr cells. Cell growth was analyzed by SRB assay after drug treatment for 72 h. (b) The combinational effects of herceptin and curcumin on BT-474 and SK-BR-3-hr cells were analyzed by trypan blue exclusion assay after drug treatment for 72 h.

Figure 3(a) shows the proliferation of different cell lines after treatment with a combination of herceptin and curcumin. In the HER-2-overexpressed BT-474 cells, we found that the antiproliferative effect of herceptin was not affected by the addition of curcumin. Curcumin could further decrease the proliferation of BT-474 cells when combined with herceptin. No apparent synergistic effect was presented when herceptin was combined with curcumin in the SK-BR-3-hr cells. The antiproliferative effect was mainly from curcumin, and the combination of herceptin with curcumin did not reveal a more antiproliferative effect than curcumin alone.

Trypan blue exclusion assay confirmed that both herceptin and curcumin inhibited the BT-474 cells dose-dependently. The combination of herceptin and curcumin further effectively decreased the cell viability of BT-474 cells (Figure 3(b)). The SK-BR-3-hr cells that we used here were resistant to herceptin but sensitive to curcumin. The combination of herceptin and curcumin effectively decreased the cell viability of SK-BR-3-hr cells. However, no apparent synergistic or antagonistic effect was present in SK-BR-3-hr cells when a combination of herceptin and curcumin was used.

The combination effect of herceptin and curcumin on the growth of BT-474 breast cancer cells was further analyzed with Calcusyn software for calculation of the CI. The combination of herceptin with curcumin exerted a biphasic interaction in BT-474 cells. The CI was less than 1 and showed a synergistic effect in the following conditions: a low dose of herceptin (0.1–1 *μ*g/mL) with curcumin (5–15 *μ*g/mL), 2 *μ*g/mL herceptin with curcumin (5–10 *μ*g/mL), or 5 *μ*g/mL herceptin with curcumin 5 *μ*g/mL. The CI was larger than 1 and showed an antagonistic effect when using a high dose of herceptin (>10 *μ*g/mL) with curcumin (5–15 *μ*g/mL). The CI and interaction between herceptin and curcumin in BT-474 cells are summarized in Table 1.

### 3.3. Effect of Herceptin and/or Curcumin on HER-2-Related Akt and MAPK Pathways in BT-474 and SK-BR-3-hr Cells

**Figure 4:**
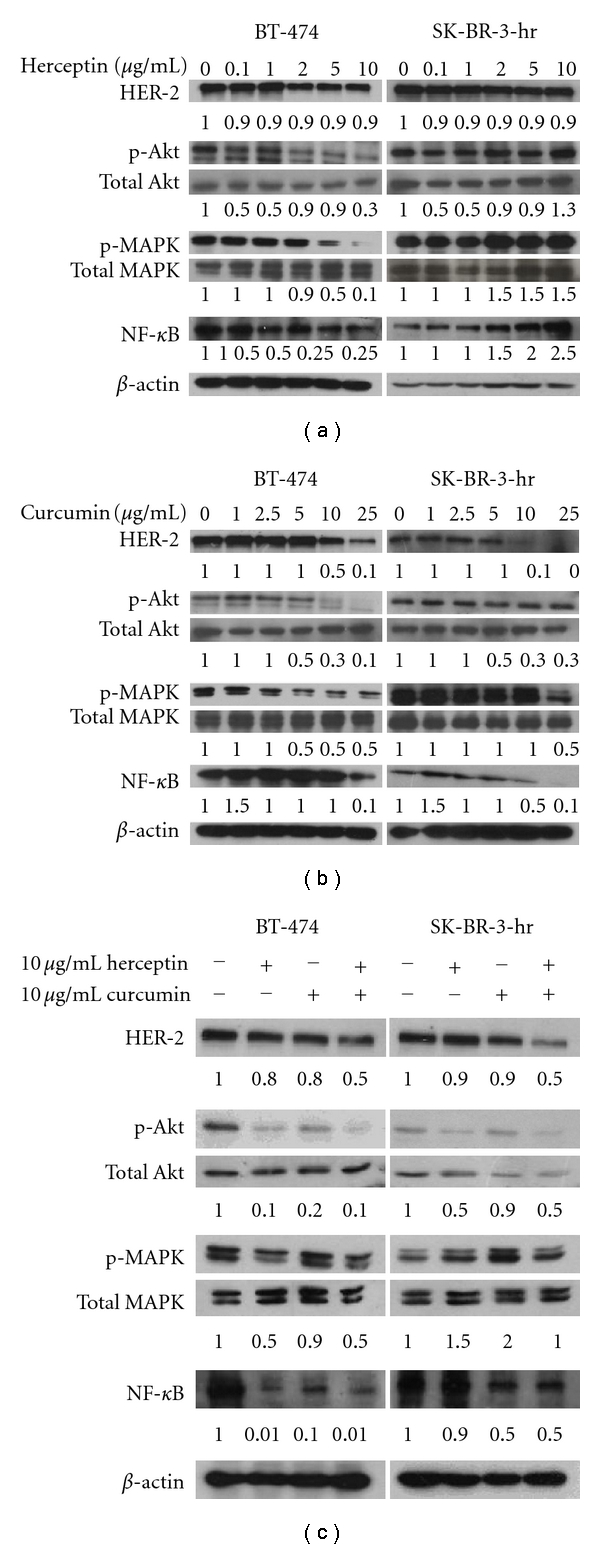
The level of HER-2, phosphorylated Akt, MAPK, and NF-*κ*B after treatment with herceptin and/or curcumin in HER-2-overexpressed breast cancer cells. The dosage effects of herceptin (a), curcumin (b), and the combination of herceptin and curcumin (c) on the BT-474 and SK-BR-3-hr cells, respectively. The number at the bottom of each lane indicates the relative fold change of control.

In the HER-2-overexpressed BT-474 and SK-BR-3-hr cells, phosphorylation of Akt and MAPK was observed (Figure 4). Herceptin dose-dependently inhibited the phosphorylation of Akt and MAPK in BT-474 breast cancer cells (left panel, Figure 4(a)). HER-2 oncoprotein was not depleted by herceptin treatment, even at high concentrations (10 *μ*g/mL). The addition of herceptin on SK-BR-3-hr cells did not decrease the expression of HER-2 oncoprotein nor decrease the phosphorylation of Akt and MAPK, even at a 10 *μ*g/mL concentration (right panel, Figure 4(a)). This was compatible with the fact that this SK-BR-3-hr cell line was a strain resistant to herceptin. When treated with curcumin, the phosphorylation of Akt and MAPK was decreased, combined with the downregulation of HER-2 oncoprotein, in a dose-dependent manner in both the BT-474 and SK-BR-3-hr cells (Figure 4(b)).

The level of NF-*κ*B in BT-474 cells was decreased along with the decreased phosphorylation of Akt and MAPK after treatment with herceptin. The level of NF-*κ*B was increased, even in high concentrations of herceptin-treated SK-BR-3-hr cells. When treated with curcumin, the level of NF-*κ*B was decreased in a dose-dependent manner in both BT-474 and SK-BR-3-hr cells. The combination of herceptin and curcumin treatment resulted in a decreased level of HER-2 oncoprotein, p-Akt, p-MAPK, and NF-*κ*B in both BT-474 and SK-BR-3-hr cells (Figure 4(c)).

### 3.4. Effect of Curcumin and/or Herceptin on BT-474 Breast Cancer Cell Cycle

To further characterize the effects of curcumin and/or herceptin on breast cancer cell growth, analyses of cell cycle phase distribution were conducted. The cell cycle of BT-474 cells without drug treatment showed a G0/G1 phase of 74%, S phase of 19%, and G2/M phase of 7%. When treated with herceptin, the G0/G1 phase increased from 74% to 79%, the S phase decreased from 19% to 11%, and the G2/M phase increased from 7% to 10%. When BT-474 cells were treated with curcumin, the G0/G1 phase remained at 74%, the S phase decreased from 19% to 9%, and G2/M increased from 7% to 20%. The combination of 1 *μ*g/mL of herceptin with 10 *μ*g/mL curcumin showed a decrease in the S phase (from 19% to 12%) without an apparent change in the G0/G1 (from 74% to 78%) and G2/M phases (7% to 10%). The results indicated that no significant change in cell cycle progression was observed in BT-474 cells after treatment with herceptin and/or curcumin.

In SK-BR-3-hr cells, the cell cycle was not changed after treatment with herceptin, which is compatible with the resistance of this cell line. When treated with curcumin for 48 h, the cell cycle of SK-BR-3-hr showed a decrease in the G1 phase (65% to 37%) and an increase in the S phase (28% to 37%) and G2/M phase (6% to 25%). Compared with the control, the combination of 1 *μ*g/mL herceptin and 10 *μ*g/mL curcumin showed a decrease in the G1 (65% to 53%), no apparent change in the S phase (28% to 27%), and an increase in the G2/M phase (6% to 19%).

### 3.5. Effect of Curcumin and/or Herceptin on the Mobility of Different Breast Cancer Cells

**Figure 5:**
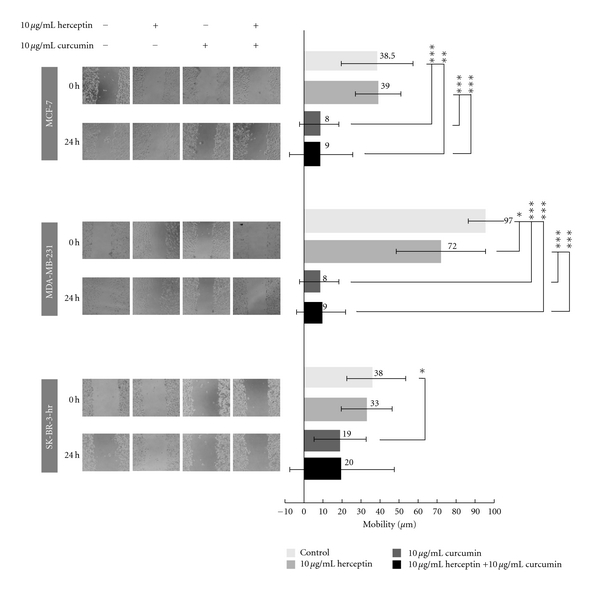
Changes in mobility of MCF-7, MDA-MB-231, and SK-BR-3-hr cells after treatment with herceptin and/or curcumin. The antimobility effect of herceptin and/or curcumin on breast cancer cells were tested by a wound-healing assay. The anti-mobility effect was illustrated in the MCF-7, MDA-MB-231 and SK-BR-3-hr cells, respectively. *P < 0.05; **P < 0.01; ***P < 0.001.

The inhibitory effect of herceptin and/or curcumin on different breast cancer cell lines was tested by wound-healing assay. Curcumin showed an apparent antimobility effect as illustrated in the MCF-7, MDA-MB-231, and SK-BR-3-hr cells (Figure 5). No effect of herceptin and/or curcumin on the mobility of BT-474 cells was observed due to the lack of migration of these cells. In the SK-BR-3-hr cells, cell migration persisted despite treatment with herceptin, which is consistent with herceptin resistance. When treated with curcumin, cell migration was greatly inhibited as compared with the control, revealing that curcumin had an apparent antimobility effect on SK-BR-3-hr cells. The combination of herceptin and curcumin did not have a better antimobility effect than curcumin alone. In the wound healing assay, curcumin showed an apparent antimobility effect in MCF-7, SK-BR-3-hr, and MBA-MB-231 breast cancer cells.

### 3.6. Curcumin and/or Herceptin Inhibited Tumor Growth in the Xenograft Animal Model

**Figure 6:**
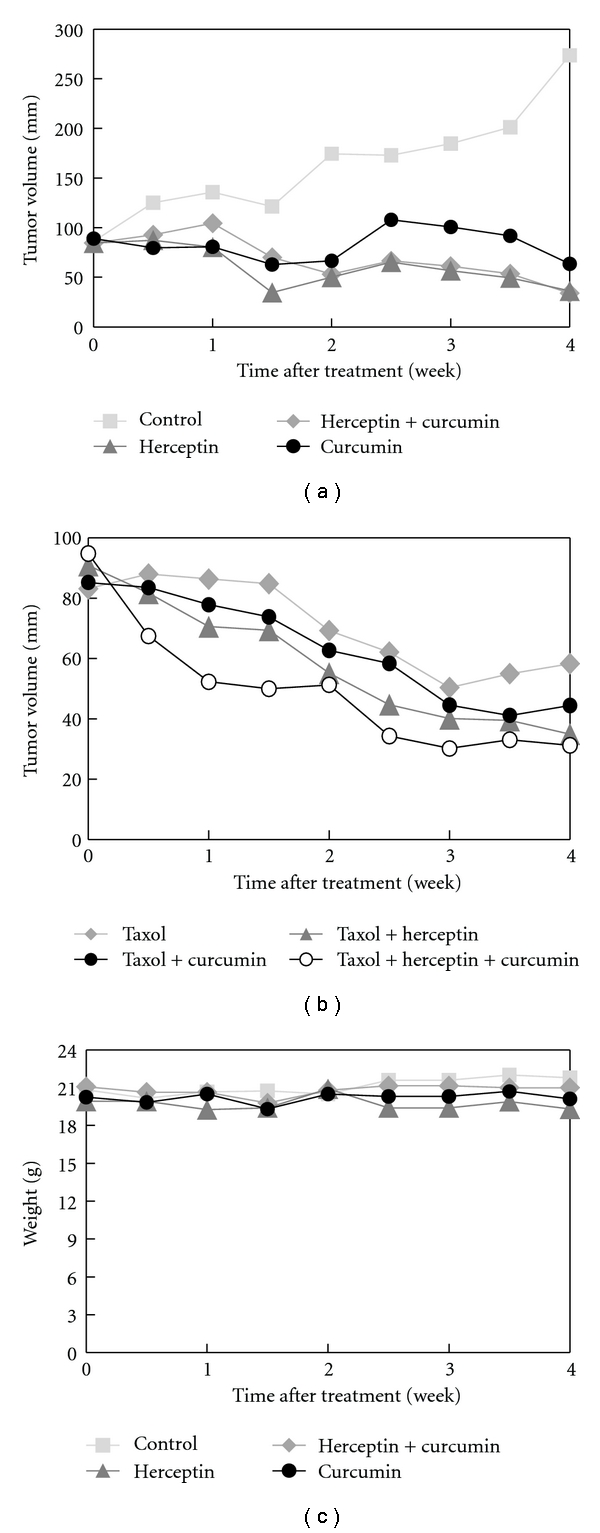
*In vivo* effects of curcumin on the herceptin and/or taxol-treated HER-2-overexpressed breast cancer xenografts. The HER-2-overexpressed BT-474 cells were injected in 4–6-week-old, female, athymic, nude mice subcutaneously at 1 × 10^7^ cells/tumor in the right flank region to form xenografts. Six mice per group were treated with different protocols with tumor volume monitored biweekly for consecutive 4 weeks. (a) The mean xenograft tumor volume change of control (0.1% DMSO), herceptin, curcumin, and combined curcumin and herceptin. (b) The mean xenograft tumor volume of taxol, taxol + herceptin, taxol + curcumin, and combined taxol + herceptin + curcumin. (c) The body weight change of these mice treated with herceptin and/or curcumin. DMSO, dimethyl sulfoxide.

Compared with the control group, the mice treated with curcumin, herceptin, or combined herceptin and curcumin all had a smaller mean xenograft tumor volume after 4 weeks of treatment (control group 273.6 ± 190.1 mm^3^, curcumin group 63.6 ± 25.7 mm^3^, herceptin group 36.3 ± 7.8 mm^3^, and combined herceptin and curcumin group 34.1 ± 25.0 mm^3^, *P* = 0.001) (Figure 6(a)). The curcumin alone group had a larger mean xenograft tumor size than the herceptin-alone group (63.6 ± 25.7 mm^3^ versus 36.3 ± 7.8 mm^3^, *P* = 0.003). The combined herceptin and curcumin group showed a smaller mean tumor volume than the curcumin-alone group (34.1 ± 25.0 versus 63.6 ± 25.7 mm^3^, *P* = 0.079) or herceptin-alone group (34.1 ± 25.0 versus 36.3 ± 7.8 mm^3^, *P* = 0.324), although without statistical significance.

The effect of combining curcumin and/or herceptin with taxol was tested by comparing the effects of taxol, taxol + herceptin, taxol + curcumin, and taxol + herceptin + curcumin regimens in the BT-474 xenograft model. In another set of experiments, 24 mice inoculated with 1 × 10^7^ BT-474 cells were randomized into 4 groups and treated with taxol, taxol + herceptin, taxol + curcumin, or combined taxol + herceptin + curcumin. After 4 weeks of treatment, the mean xenograft tumor volumes were taxol 58.3 ± 11.2 mm^3^, taxol + herceptin 35.0 ± 13.4 mm^3^, taxol + curcumin 44.5 ± 6.2 mm^3^, and combined taxol + herceptin + curcumin group 31.3 ± 27.7 mm^3^. The antitumor effect of taxol + herceptin was apparent, and combined taxol and curcumin had a comparable antitumor effect (44.5 ± 6.2 versus 35.0 ± 13.4 mm^3^, *P* = 0.884). The combination of taxol, herceptin, and curcumin resulted in the smallest tumor volume, but this was not statistically different from that of the taxol and herceptin regimen (31.3 ± 27.7 versus 35.0 ± 13.4 mm^3^, *P* = 0.079) (Figure 6(b)). The body weight of these mice treated with herceptin and/or curcumin was quite stable during the 4-week period (Figure 6(c)).

## 4. Discussion

This study was designed to test the efficacy of curcumin in HER-2-overexpressed breast cancer, with a direct comparison with herceptin in the *in vitro* cell line and *in vivo* xenograft animal model. Our preclinical result revealed that curcumin reduced the cell viability of different breast cancer cell lines, including MCF-7 (ER-positive, HER-2-negative), MDA-MB-231 (ER-negative, HER-2-negative), HER-2-overexpressed BT-474 (ER-positive, HER-2-positive), and herceptin-resistant SK-BR-3-hr (ER-negative, HER-2-positive) cells. The level of HER-2 oncoprotein, p-Akt, p-MAPK, and NF-*κ*B were decreased in a dose- and time-dependant manner in BT-474 and SK-BR-3-hr cells when treated with curcumin. The cell cycle perturbation by curcumin was mainly found in the increase in the G2/M phase. The apparent antimobility effect of curcumin was also revealed in the MCF-7, MDA-MB-231, and SK-BR-3-hr cells. The combinational effect of herceptin with curcumin was a biphasic interaction on the growth of BT-474 cells. When a low dose of herceptin was used with curcumin, there was a synergistic effect, but an antagonistic effect was observed when a high dose of herceptin was used. In the BT-474 xenograft model, curcumin treatment effectively decreased the tumor size, and the combination of taxol with curcumin had an antitumor effect comparable with that of taxol and herceptin treatment.

The action of herceptin in inhibiting ErbB2 (HER-2) signaling involves the reduced phosphorylation of Akt but not endocytic downregulation of ErbB2 [42]. HER-2 oncoprotein was not depleted by herceptin, even at a high concentration (Figure 4). The exact mechanism of herceptin resistance is not clear, and possible mechanisms include obstacles to herceptin-binding to HER-2, upregulation of HER-2 downstream signaling pathways, signaling through alternative pathways, or failure to trigger immune-mediated mechanisms to destroy tumor cells [14]. In the herceptin-resistant SK-BR-3-hr cells, the persistent activation of PI_3_K/Akt and MAPK pathways, despite treatment with herceptin, may have been acquired through repeated cultures of herceptin-treated SK-BR-3 cells.

NF-*κ*B activation played an important role in chemotherapy resistance [29, 30], and targeting NF-*κ*B showed improved benefits in some preclinical HER-2-overexpressed cancer cells [28, 43]. In HER-2-overexpressed breast cancer, ErbB2 activates NF-*κ*B via signaling that includes PI3K, PDK1, Akt, protein kinase 2 (CK2), and CKBBP1 [44]. In herceptin-sensitive HER-2-overexpressed BT-474 cell lines, herceptin treatment effectively decreased the phosphorylation of Akt, MAPK and the expression of NF-*κ*B (Figure 4). Whether the resistance to herceptin was also related to NF-*κ*B activation is not clear. In the herceptin-resistant SK-BR-3-hr cells, the expression level of NF-*κ*B increased in parallel with the incremental dose of herceptin. When treated with curcumin, the expression of NF-*κ*B was decreased, accompanied with increased cell death. The ability of curcumin to inhibit herceptin-resistant SK-BR-3 cells may be related to the downregulation of HER-2 oncoprotein, and suppression of related Akt, MAPK, and NF-*κ*B signaling pathways. The ability of curcumin to downregulate EGFR and HER-2 oncoproteins and inhibit the phosphorylation of Akt and MAPK and NF-*κ*B activation suggests that curcumin has potential in the treatment of HER-2-overexpressed and/or herceptin-resistant breast cancer.

The interaction of curcumin and herceptin in HER-2-overexpressed cancer has rarely been reported. Whether the combination of herceptin with curcumin had any therapeutic advantage over herceptin alone is unknown. In the *in vitro* BT-474 cell line study, the combination of herceptin and curcumin showed an advantage over either treatments alone (Figure 3). The combination of herceptin and curcumin exerted a biphasic interaction on the growth of BT-474 cells. A synergistic effect was present when a low dose of herceptin (0.1–1 *μ*g/mL) was combined with curcumin, while a high dose (>10 *μ*g/mL) of herceptin would exert an antagonistic effect when combined with curcumin (Table 1). In the SK-BR-3-hr cells, the combination of herceptin with curcumin exerted neither a synergistic nor antagonistic effect. This biphasic interaction observed *in vitro* warrants caution when herceptin is to be used with curcumin or other medications.

In the xenograft animal study, curcumin treatment effectively reduced the tumor volume by 76.7%, compared with the control; however, it was not as effective as of herceptin, which achieved an 86.7% tumor reduction (Figure 6(a)). The combination of herceptin and curcumin showed a greater antitumor effect than curcumin alone (87.5% versus 76.7% in tumor regression) but a similar effect to that of herceptin (87.5% versus 86.7%). The anticipated synergistic effect of combining herceptin with curcumin was not observed in our *in vivo* xenograft animal model. Although curcumin might not interfere with herceptin in the normal physiologic concentration, from our* in vivo* study, combined herceptin and curcumin was not better than herceptin alone. More solid evidence might be needed to support the rationale of combing herceptin with curcumin in the treatment of HER-2-overexpressed breast cancer.

It has been reported that curcumin could suppress taxol-induced NF-*κ*B, and curcumin combined with taxol showed greater antitumor effects than taxol alone [31, 33]. In our* in vivo* xenograft study, the combination of curcumin and taxol had therapeutic effects comparable with taxol and herceptin, one of the current preferred regimens for HER-2-overexpressed breast cancer (Figure 6(b)). The combination of taxol + herceptin + curcumin was associated with the smallest mean tumor volume although this was not statistically different from that of the taxol and herceptin regimen.

Curcumin was rated safe and well-tolerated [45]; however, the application of curcumin might be limited by its low bioavailability [16, 17, 45, 46]. To reduce the impact of the low bioavailability of oral intake, we used an intra-peritoneal injection to treat xenograft nude mice. In our study, the body weight of herceptin and curcumin-treated mice did not vary greatly during the entire treatment period (Figure 6(c)), demonstrating the relative safety and tolerability of curcumin as previously reported in animal and human studies [16, 47, 48]. The main limitation of our *in vivo *study was that we had only one fixed dose protocol for herceptin and/or curcumin treatment, and the serum concentration of the drugs was unknown. The dose of herceptin used here was in accordance with the current practice guideline [26], while the dose of curcumin was derived from a previous report [49]. The optimal dose of curcumin for HER-2-overexpressed breast cancer is unclear and needs to be determined for maximum therapeutic effect.

In this study, we showed that curcumin could reduce the cell viability of both HER-2-overexpressed herceptin-sensitive BT-474 cells and herceptin-resistant SK-BR-3-hr breast cancer cells. In the BT-474 xenograft model, though not as much as herceptin, curcumin did effectively decrease the tumor size. The combination of curcumin with herceptin was not better than herceptin alone; however, the combination of taxol and curcumin had an antitumor effect comparable with taxol and herceptin. The results, both *in vitro* and *in vivo*, suggested that curcumin has the treatment potential for HER-2-overexpressed breast cancer.

## Figures and Tables

**Table 1:** The combination index of herceptin and curcumin treatment of the growth of BT-474 cells.

Herceptin (*μ*g/mL)	Curcumin (*μ*g/mL)	Fa	CI	Effect
0.1	5	0.34	0.669	Synergistic
0.1	10	0.45	0.836	Synergistic
0.1	15	0.54	0.900	Synergistic
1	5	0.52	0.393	Synergistic
1	10	0.52	0.713	Synergistic
1	15	0.54	0.953	Synergistic
2	5	0.52	0.467	Synergistic
2	10	0.53	0.750	Synergistic
2	15	0.54	1.013	Additive
5	5	0.5	0.794	Synergistic
5	10	0.52	1.007	Additive
5	15	0.56	1.075	Additive
10	5	0.49	1.354	Antagonistic
10	10	0.54	1.195	Antagonistic
10	15	0.57	1.241	Antagonistic

A value of CI <1 represents a case where synergism of herceptin and curcumin was present. CI values of 1 and >1 represent additive and antagonistic effects, respectively. Fa, fraction affected; CI, combination index.
